# Medical school curriculum in the digital age: perspectives of clinical educators and teachers

**DOI:** 10.1186/s12909-022-03454-z

**Published:** 2022-06-03

**Authors:** Humairah Zainal, Xiaohui Xin, Julian Thumboo, Kok Yong Fong

**Affiliations:** 1 Health Services Research Unit, Singapore General Hospital, SingHealth Tower Level 16, 10 Hospital Boulevard, Singapore, 168582 Singapore; 2 Department of Rheumatology and Immunology, Singapore General Hospital, SingHealth Tower Level 16, 10 Hospital Boulevard, Singapore, 168582 Singapore; 3 Duke-NUS Medical School, National University of Singapore, Singapore, Singapore

**Keywords:** Clinical competence, Technology, Medical education, Curriculum, Singapore

## Abstract

**Background:**

There is a need to reexamine Singapore’s medical school curricula in light of the increasing digitalization of healthcare. Notwithstanding Singapore’s digital competitiveness, there is a perceived gap in preparing its medical students for the digital age. Furthermore, limited research has evaluated the extent to which skills in using digital technologies should be taught to medical students in Asian medical schools to prepare them for future clinical practice- a gap that is filled by this study. Using Singapore as a case study, it explores the views of some local clinical educators and teachers towards the need to impart skills in digital technologies to medical students. It also offers recommendations on ways to balance the clinicians’ concerns about these technologies with the digital competencies needed for clinical practice.

**Methods:**

Findings were drawn from individual interviews with 33 clinical educators and teachers from Singapore’s public and private healthcare sectors. They were recruited using purposive sampling. Data were interpreted using qualitative thematic analysis.

**Results:**

Participants included vice deans of education from all three local medical schools and senior consultants from a wide variety of disciplines. Overall, they acknowledged two benefits of equipping students with skills in digital technologies including promoting the culture of innovation and improving work efficiency. However, they also highlighted four main concerns of imparting these skills: (i) erosion of basic clinical skills, (ii) neglect of a generalist approach to healthcare characterized by holistic management of patients, inter-professional collaboration, and commitment to breadth of practice within each specialty, (iii) rapid pace of technological advances, and (iv) de-personalisation by technology.

**Conclusions:**

The findings show that medical students in Singapore would benefit from a curriculum that teaches them to use digital technologies alongside core clinical skills.

## Background

Singapore is a frontrunner in digital transformation. The Global Connectivity Index (GCI) 2020 ranked Singapore second after the United States in terms of enhancing user experience and prioritizing investments in 5G, big data, Artificial Intelligence (AI) and Internet of Things (IoT) [1]. In the context of healthcare, the COVID-19 pandemic and an increasingly ageing population have accelerated the adoption of digital technologies such as AI, robotics and telemedicine in Singapore’s healthcare system [2, 3]. Despite these advances, there is a perceived gap between the digital skills taught in medical schools and those deemed useful by medical students and junior doctors for clinical care [4]. This gap is not unique to Singapore, as studies from other developed countries such as the United States and in Europe have also reported similar trends in the education of digital technologies [5–7]. Such technologies would include telemedicine, extended reality, AI, machine learning, simulations, personalized medicine and genomics, which were listed by Forbes as the biggest technology trends that will transform medicine and healthcare in 2022, and which would be the focus of this article [8].

Medical schools need to consider equipping students with the necessary digital competencies for them to succeed in clinical practice, as there is currently a disconnect between contemporary practice and medical school training, which is still largely memorization-based [9, 10]. Furthermore, incorporating digital competencies into the core curriculum would equip future medical graduates with the knowledge of how to utilise medical data, digital infrastructures in the health system, digital technologies used in patient care, of the medico-legal and ethical aspects of using these technologies, as well as other digitalization processes [11]. Moreover, one should not assume that students who grow up in the digital era are technically competent in digital technologies, as technological savviness does not necessarily translate into competence in using digital technologies in patient care [12–14].

Of note, several barriers to implementation exist including institutional inertia, faculty resistance to curricula change, inconsistent availability of technological resources for medical education, and a densely packed medical curricula [15–17]. Despite their significant role in medical education and in the healthcare system, few research has explored the views of clinical educators and teachers in addressing this conundrum. Most original studies have focused on the views of medical students and junior doctors towards their knowledge and confidence level of using specific digital technologies in healthcare [12, 14, 18, 19]. Our study fills these gaps by exploring the views of 33 senior clinical educators and teachers in Singapore towards the integration of skills in digital technologies within the core medical school curriculum. It builds on the extant literature by examining the potential benefits and challenges of teaching these technologies in the context of core clinical skills, such as physical examination, history-taking, clinical reasoning and communication, which are fundamental to the art and science of doctoring. Hence, this article bears important implications for medical schools in developed countries that are evaluating the extent to which they should impart skills in using digital technologies to medical students.

## Methods

### Sample and setting

The consolidated criteria for reporting qualitative research (COREQ) was followed in the reporting of this study [20] (Table 1). Data was collected from October 2020 to January 2021 through semi-structured interviews with clinical educators and teachers. For maximum variation, purposive sampling was used to recruit vice deans of education from all three local medical schools, and senior consultants who are also clinical educators and clinical teachers from different surgical and medical specialties such as vascular surgery, cardiology and dermatology. The latter were recruited from academic teaching hospitals in the public sector, a private family medicine clinic and the Singapore Ministry of Health. The participants were recruited through email invitations. Waiver for ethical approval was granted by SingHealth Centralised Institutional Review Board (Reference Number: 2020/2880).

**Table 1:** COREQ (COnsolidated criteria for REporting Qualitative research) Checklist

Topic	Guide Questions/Description
Interviewer/facilitator	HZ
Credentials	HZ [Ph.D.], XX [M.Soc.Sci], JT [MBBS, MMed (Internal Medicine), FAMS (Rheumatology), FRCP] and FKY [MBBS, MMed (Internal Medicine), FAMS (Internal Medicine, Rheumatology), FRCP]
Occupation	HZ- Research Fellow, XX- Senior Research Manager, FKY and JT- Senior Consultant, Rheumatology and Immunology
Gender	HZ- female, XX- female, JT- male, FKY- male
Experience and training	HZ- Trained in qualitative research and methodology, educational background in Sociology. XX- educational background in Sociology. JT- educational background in Medicine. FKY- educational background in Medicine
Relationship established	No
Participant knowledge of the interviewer	Participants were informed about the study by the Principal Investigator through email. The research fellow of this study then contacted the participants to schedule for an interview
Interviewer characteristics	No other characteristics were reported by the interviewer
Methodological orientation and Theory	Coding frameworks and themes were developed iteratively using Braun and Clarke’s (2006) six-step process which include: (i) familiarizing ourselves with the data, (ii) generating initial codes, (iii) searching for themes, (iv) reviewing themes, (v) defining and naming the themes, and (vi) producing the report. With regard to the themes generated under part (iii), please refer to the section on ‘clarity of major themes’ and ‘clarity of minor themes’ in this table
Sampling	Purposive sampling and snowballing
Method of approach	Via email
Sample size	33 participants
Non-participation	Not applicable
Setting of data collection	30 were done over Zoom (due to the physical and social restrictions brought about by the COVID-19 pandemic) and 3 were done in-person (due to interviewees’ preference)
Presence of non-participants	None
Description of sample	Male = 78.8%, female = 21.2%, clinical educator = 66.7%, clinical teacher = 33.3%
Interview guide	Refer to Table 2
Repeat interviews	None
Audio/visual recording	Zoom and audio recording
Field notes	Yes, made after interview to jot down fieldwork impressions
Duration	Approximately 40 min each
Data saturation	Yes
Transcripts returned	No
Number of data coders	Two
Description of the coding tree	Yes. Under ‘Data Analysis’ in the manuscript
Derivation of themes	Inductive, derived from the data
Software	Nvivo 12
Participant checking	No
Quotations presented	Yes
Data and findings consistent	Yes
Clarity of major themes	Yes. These include the types of digital competencies relevant to the digital age, improvements to be made to current curricular content, and benefits and concerns of imparting these skills for clinical practice and acumen
Clarity of minor themes	Yes. These include interviewees’ perceptions of young doctors’ digital competencies, as well as role of professional bodies, healthcare institutions and healthcare system

### Data collection

Written informed consent was obtained from all the participants who participated in the study. All the interviews were conducted in English. 30 were conducted over Zoom whereas three were done in person. Each interview lasted approximately 40 min. The interview questions included relevant skills that would guide the clinical practice of future medical graduates in the digital age, and other improvements that can be made to the core medical school curriculum to better prepare them for increasing digitalization of healthcare (Table 2).

**Table 2:** Interview Questions

No	Questions
1	In general, what are the clinical skills that a medical doctor should have?
2a	Which of these skills are still relevant in the digital age?
2b	Are there any skills that have been replaced by digital technology, be it partially or completely?
3a	What new skills, clinical or otherwise, should a doctor have today and in the future in order to practise medicine?
3b	In your opinion, are our locally trained doctors well-equipped with these skills?
4a	What clinical skills are currently being covered in the local medical schools?
4b	Which of these skills should be emphasised more in the medical school curriculum?
5	What other improvements can be made to our local medical school curriculum to better prepare the students for clinical practice in light of rapid advances of technology (for example, the advent of Artificial Intelligence, big data, imaging, smartphone applications, and digital equipment such as handheld ultrasound)?
6	How can local medical schools improve their collaborations with professional bodies and healthcare institutions to prepare medical students for clinical practice in this era of new technology?
7	What can the healthcare system do to support medical students and young doctors in this era of new technology?
8	How can digital technology aid doctors’ clinical practice and acumen?
9	Do you have any other comments on the digital transformations of medicine or healthcare before we end this interview?

During the interview, the researcher briefed participants about the study before obtaining their verbal consent to record the session. A common list of digital technologies based on Forbes’ latest technology trends were also mentioned to trigger discussions [8]. The interviews were then transcribed verbatim by a transcriber. The transcripts were reviewed by the researcher and Principal Investigator to ensure transcription accuracy. In reviewing issues of reflexivity in qualitative research [21], we were made aware of potential researcher bias that may be present due to the established professional relationship between the Principal Investigator and research participants. The interviewer, having no prior relationship with any of the participants, is an important measure to counter the threat of this bias. To prevent this from influencing our research, we assigned code identifiers to each participant to ensure anonymity. Codes that begin with ‘SC’ refer to senior consultants who are clinical teachers whereas those that start with ‘ED’ refer to clinical educators.

### Data analysis

The researcher and Principal Investigator read the transcripts independently and adopted an inductive thematic analysis approach when evaluating the data to draw common and shared meanings among participants [22]. Coding frameworks and themes were developed iteratively using Braun and Clarke’s (2006) six-step process [23]. Data from senior doctors trained in different specialties and who were working in different healthcare and educational institutions were collected to ensure accuracy of results. We also compared the findings with current local and global literature on medical education trends for future doctors in the era of digital technologies [3, 6, 24–26]. Any coding discrepancies were resolved through consensus between the researcher and Principal Investigator, and through seeking the opinions of our co-authors.

## Results

Thirty-three senior clinical educators and teachers from 24 different disciplines as well as 12 healthcare institutions, three medical schools, one private clinic and the Ministry of Health participated in the study (Table 3). All of them have more than 10 years of clinical experience, and are aged between 44 and 70 years old.

**Table 3:** Demographics of Participants

Roles	Clinical educator^a^	Clinical teacher^b^
Number of participants	22	11
**1 interviewee per discipline**	**2 interviewees per discipline**	**3 interviewees per discipline**
Cardiology Dermatology Ear, Nose and Throat Surgery Endocrinology Gastroenterology and Hepatology Hepatobiliary Surgery Infectious Diseases Medicine Internal Medicine Medical Oncology Neurology Obstetrics and Gynaecology Ophthalmology Pathology Public Health Radiology Renal Medicine Respiratory and Critical Care Medicine Vascular Surgery	Anaesthesiology Geriatrics Medicine Paediatrics Medicine	Emergency Medicine Family Medicine Rheumatology

^a^A clinical educator has an interest in teaching and generally spends more than 20 per cent of the working week in education-related matters such as bedside teaching, curriculum planning, education faculty administration and education research

^b^A clinical teacher has an interest in teaching and generally spends less than 20 per cent of the working week in education-related matters

Overall, they acknowledged the benefits of teaching medical students some digital skills which include the following:

### Promote the culture of innovation among students

Some of the interviewees believed that sufficient exposure to new technologies in medical school would help to instill the spirit of research and innovation among medical students, which would help pave the way for them to make major breakthroughs in the future:

If we have a module on robotics for medical students, that will be very interesting for various reasons. One, is to introduce the field to them. Two, is to instill the culture of innovation among students. And if we were to put more emphasis on Research and Development in the curriculum, students will not only embrace the technology; they will also be encouraged to develop and pioneer something new on their own. [SC-22, ENT Surgery]

Indeed, medical students in Singapore have had limited exposure to research and innovation opportunities, though this has changed recently. For example, it was only in 2017 that a student-led medical innovation programme was introduced by the Yong Loo Lin School of Medicine at the National University of Singapore (NUS). Known as the NUS Medical Grand Challenge, the programme teams up medical students with students from two other faculties to solve unmet healthcare needs within a year [27]. Even so, the projects may not necessarily involve experimentation with digital technologies. Moreover, students would only have encounters with telemedicine during their rotations in family medicine clinics, which have a duration of several weeks for some schools [28]. Current initiatives to encourage research and innovation among students are also not applied uniformly in the core curriculum of all medical schools. As it stands, only students of Duke-NUS Medical School which offers a four-year graduate programme, have a dedicated year to embark on a research project.

### Improve work procedures and efficiency

Another rationale for imparting skills in using digital technologies lies in the need to enhance work efficiency and ensure safe work procedures, as shared by an interviewee below:

The increasingly digital world is meant to help the doctors, hopefully, by reducing errors through checks. So, for example, nowadays, you may not even need to ask patients about their diabetic control because it’s all in the system. But it still requires you to have basic clinical skills to ensure that the digital system helps make things safer. [SC-03, Ophthalmology]

Being equipped with ‘basic clinical skills’ in the context of digital technologies entails several sets of skills, as shared by other participants. These include knowing how to respect patient privacy and confidentiality when using digital platforms (ED-01, ED-04) and being trained in the ethical aspects of managing digital technologies in ways that lead to improved patient outcomes and safety (ED-13). These skills however, have not been intentionally taught in the local medical school curriculum. In relation to these, the participants also shared four key concerns of introducing digital competencies in the medical school curricula, as cited below.

### Erosion of basic clinical skills

Most interviewees were concerned that training in digital technologies may lead to the erosion of basic clinical assessment skills such as physical examination and an over-reliance on imaging, scans and laboratory test results. Additionally, basic clinical skills were perceived to be important when practising in diverse settings, as illustrated by the excerpts below:

I would be very sad if we don’t need to listen to hearts or examine abdomens anymore because you could scan everything and get the answers to that. But even in the digital age, these skills must be emphasised because we don’t want to create an over-reliance on imaging or other tests. We also have to plan for practice in different settings where this technology may not be readily available and we would be extremely handicapped. [ED-07, Rheumatology]

If we want to produce doctors who are globally adaptable, we cannot afford to allow digitalization in terms of training. Practice is fine. At the school level, we should still achieve the basic skills. I do overseas missions in villages in the Philippines and India. A lot of times, there are no facilities. We just have to use our stethoscopes and pick the best of what we can hear. So, we still need to teach students basic skills so that we don’t embarrass ourselves in the multinational relief teams, for example. [SC-17, Gastroenterology and Hepatology]

Basic clinical skills, particularly diagnostic and therapeutic procedures, have been outlined by The National Medical Undergraduate Curriculum Committee as competencies that a medical graduate must be proficient in upon graduation [29]. Hence, schools should not lose sight of these skills as they train students to pick up new digital skills. Moreover, as underscored by the interviewees, it is important to train students to be highly adaptable and flexible in the digital age. Surrounded by many developing countries that may not have state-of-the art healthcare equipment, doctors who are assigned to overseas humanitarian missions are expected to make use of their foundational skills in the absence of such equipment.

### Neglect of a generalist approach

Another concern if specific technologies were to be taught to students pertains to the potential neglect of a generalist healthcare approach, a philosophy of care characterized by the holistic management of patients, a commitment to the breadth of practice, and collaboration within the larger healthcare team:

A problem we’ve had for decades is that we’ve gone down this inexorable barrage of super-specialisation and lost the ability to examine patients as a whole… A danger of technology is that it allows each specialty to become even more super-specialised. The question then is, who is going to do the general holistic care of human beings? [ED-08, Vice-Dean of Education]

In this age, we are no longer practicing solo medicine anymore. The ability to collaborate widely with large groups of people from diverse professional backgrounds is also an important skill to have. Given the complex nature of medicine and the evolving landscape, we need to have a ‘systems view’ of medical issues and a broad understanding of big data and artificial intelligence. Not so much of the specifics. [ED-10, Oncology]

Based on the above excerpts, exercising a broad practice set rather than focusing on niche areas of sub-specialties that often operate in silos is preferred, as it allows doctors to respond more effectively to the needs of the patient and the community. This has also been proven by studies that reiterate the importance of keeping a broad framework in the advancement of the traditional curriculum and of aligning the curriculum with the needs of the healthcare systems in which students will practice [30, 31].

### Rapid pace of technological advances

The rapid evolvement of digital technologies forms the concern of some interviewees, who shared that it would be more practical to teach students the fundamental concepts, principles and applications behind the technologies than the specificities:

It’s better to teach students emerging technologies as a concept and principle because who knows if they won’t be relevant in two- to five-years’ time. [ED-08, Vice-Dean of Education]

It’s more important for students to be able to appreciate the digital formats and concepts behind AI, for example, what AI can do, its pitfalls, the pros and cons on a conceptual level. [ED-04, Pathology]

This concern is especially applicable to Singapore, which was ranked the world’s second most digitally competitive country after the US in the IMD World Digital Competitiveness Ranking in 2020 [1]. A major characteristic of the top performing countries is the ability to adopt new technologies quickly for economic and social transformation [1]. In view of the rapid pace of technological adoption, as well as the dynamic digital landscape, it is all the more feasible to keep the training in digital technologies broad.

### Depersonalisation by technology

Other interviewees expressed concern that digital technologies may lead to further depersonalisation of medicine:

My concern is, how do we ensure that medicine remains personal? There’s already some depersonalisation with our Electronic health record systems (EHRs). Patients are complaining that the doctor is only looking at the computer and does not maintain eye contact with them. Therefore, we need to be aware that technology may have a trade-off. [SC-01, Family Medicine]

Human interaction has always been important but unfortunately, it is not strong to begin with. The use of technology is going to erode communication skills even further. We tend to tell people to follow algorithms, policies and procedures. And then we get overloaded with a lot of electronic data to the point that there is no time to engage with patients. [SC-04, Family Medicine]

This issue is especially salient for Asian countries like Singapore, where family members are typically involved in the patient’s care and decision-making processes [32]. In addition to having good inter-personal skills in order to communicate well with both patients and their family members, doctors need to dedicate the appropriate amount of time to engage with them and attend to their needs. After all, ‘interpersonal and communication skills’, and ‘professionalism’ form part of the core competencies outlined by the Accreditation Council for Graduate Medical Education (International) (ACGME-I) [29]. With the advent of new technological tools, students need to be trained to harness digital technologies in ways that enhance their clinical competencies.

## Discussion

The findings have highlighted two perceived benefits and four main concerns of the senior clinicians with regard to equipping students with skills in digital technologies. The former include promoting the culture of innovation early in medical school and ensuring safe and efficient work processes. The interviewees acknowledged that more should be done to expose students to digital technologies and that these technologies should only be taught if they are beneficial to patients. At the same time, they raised concerns such as the erosion of basic clinical skills, neglect of a generalist approach to healthcare, rapid pace of technological advances and depersonalisation by technology. In so doing, they have called for a continuing emphasis on basic clinical skills and humanistic skills, particularly communication skills, and a reduced emphasis on sub-specialty skills and technical knowledge. In view of the rapid pace of technological advances, they have proposed the teaching of broad principles of digital technologies and systems rather than specific utilizations.

Studies performed in Canada and the US have reported similar results. In particular, they have also discussed the continuing relevance of traditional clinical skills amid increasing digitalization in healthcare [33, 34]. These include Garibaldi et. al.‘s (2017) study that demonstrates that physical examination still outperforms technology in terms of diagnosis, rapport building with patients and other important instances [33]. Rousseau et. al.’s (2018) research validates this by showing how an over-reliance on technology threatens the culture of teaching physical examination at the bedside [34]. In terms of the embodiment of holistic skills, Han et. al. (2019) have highlighted the myriad of benefits of teaching medical students inter-professional collaboration amid technological advances. These include learning how to respect others’ viewpoints and collaborate with other healthcare professionals for patients’ safety, gaining a deeper understanding of patients’ perspectives, and developing an appreciation of coordinating resources to provide the best care [26]. With regard to the rapid evolvement of technology, Aungst and Patel’s (2020) research has also shown how the short life cycle of new technologies and their ambiguous role in clinical effectiveness may pose a challenge to the establishment of educational standards for digital health [24]. The unintended consequences of using technologies such as EHRs indiscriminately on doctor-patient relations have also been reported by other studies [35].

While there has been a growing body of works on digitalization within the medical school curriculum in Western countries, there is a paucity of literature on this topic in the Asian context. Studies conducted elsewhere in Asia, such as in Hong Kong, Japan and Taiwan have largely focused on equipping students with skills in using point-of-care ultrasound (POCUS) and laparoscopic surgery [36–38]. In Singapore, laparoscopic surgery is taught to doctors only when they enter residency, as it requires specialised skills. Furthermore, unlike past studies that have mostly evaluated a digitally-integrated curriculum in the form of an elective coursework [7, 39, 40], our work discusses this integration within the context of the core curriculum in recognising the urgent need to equip Singapore’s medical students with the necessary digital competencies. In so doing, it reiterates the importance of implementing specific learning outcomes in the compulsory curriculum, as highlighted by other scholars such as Foadi et. al. (2021) and Gonzalo et. al. (2017) [11, 30].

Our study shows that equipping students with the necessary skills for the digital age involves more than just identifying gaps in the curriculum or in specific technologies. Based on the findings, it also entails considering whether the skills imparted would prepare students for clinical practice in different types of local and global settings, contextualizing the skills to the needs of society and current work environment, assessing the evolving technological landscape and ensuring that technology in general would not replace the human touch. Hence, beyond proposing recommendations for the formal curriculum, this study also calls for a consideration of how the undergraduate medical education, the clinical setting, the healthcare system and the broader digital landscape could inform one another. Additionally, any new skills introduced in the curriculum should take into consideration the ultimate risks and benefits to the patient.

### Recommendations

Findings from the interviews have reiterated the importance of balancing the interviewees’ concerns with the digital competencies needed for clinical practice. This section offers recommendations to address each of these concerns.

#### Complement basic clinical assessment skills with digital skills

The findings show that digital technologies should not be a substitute of traditional clinical skills. Instead, the former should be an adjunct to the latter, particularly in the teaching of physical examination skills. Studies have shown that a combination of teaching strategies optimizes learning and increases students’ confidence in their clinical abilities [41, 42]. However, these studies are mostly focused on the use of ultrasound, and are based on students’ self-assessment of their abilities, which lack objective measures. The way in which newer forms of digital technologies such as AI can enhance students’ competencies should also be rigorously explored by using objective and validated assessment tools.

In addition, not all specialties will use the technologies equally; some will use them more than others. According to Benjamens et. al. (2020), the vast majority of AI/ML-based technologies/algorithms approved by the United States Food & Drugs Administration (FDA) were developed for radiology (46.9%) and cardiology (25%). Only about 16% were for internal medicine and general practices [43]. Hence, more evidence is needed to determine how much digital-related skills need to be introduced in the medical school curriculum and which of these skills are better suited to be taught during the advanced years of medical education instead.

#### Develop generalist skills that are compatible with the needs of aging societies

Having a set of generalist clinical skills becomes all the more important for an ageing society with multiple morbidities, of which Singapore is an example because it has one of the fastest ageing populations in the world. Indeed, this is a need in many developed countries, as illustrated by studies done in European countries by Rijken et. al. (2018) and Søndergaard et. al. (2015), which likewise highlight the importance of holistic care and inter-professional collaboration among healthcare teams [44, 45].

To ensure that students adopt a generalist approach to healthcare, it is pertinent to ensure that the undergraduate curriculum remains broad and fundamental. Specialised skills should only be taught in the advanced years of medical training when physicians enter residency and train to become specialists. At the undergraduate level, students should be equipped with the necessary digital skills that are aimed at strengthening their capacity to practice medicine in a data-rich environment supported by AI while assuring the mastery of compassionate care and of paving improved outcomes for older patients with chronic diseases [46, 47]. In particular, they should be equipped with knowledge of the four fundamental ‘V’s of big data as identified by Wartman et. al. (2018), which include ‘volume’, ‘variety’, velocity’ and ‘veracity’ of data. Thus, medical schools play an important role in building a foundation for students to aggregate, analyse and personalize big data in health care delivery through AI applications [46].

#### Emphasize concepts and principles rather than technical knowledge

In view of the rapid advances of new technologies, teaching students the enduring concepts and principles behind these technologies rather than technical knowledge would help to ensure the retention of core skills. A focus on the former has been favoured in past studies that use technology as an adjunct to teaching basic clinical skills. For instance, Ahn’s (2015) research, which evaluates whether the addition of ultrasound to traditional physical examination instruction improves medical students’ abilities to locate the femoral pulse, demonstrates that combining diagnostic scanning with physical examination and anatomy instruction provides a more holistic curriculum and contributes to students’ overall clinical competency rather than just proficiency in ultrasound [41]. Indeed, students should not necessarily be expected to be proficient in digital technologies, as their future role would ultimately be oriented towards patient care and the clinical applications of these technologies.

Furthermore, since technological advances and the evolution of the medical school curriculum are often contemporaneous with each other, it would be unrealistic to predict all future trends and competencies relevant to students. Hence, continuing medical education would also play an important role in equipping doctors with the necessary competencies. Health systems, group practices and clinical leaders would also need to work together to provide ongoing education for practicing doctors [15, 25, 48].

#### Harnessing digital technologies to enhance clinical skills

To ensure that the human touch is not lost with the advent of technology, there should be a shift in clinicians’ attitude towards digital technologies, which should be used to complement or enhance instead of replace core clinical skills. In the context of the medical school curriculum, students can be taught ways to complement clinical decision-making processes using digital technologies such as ML with the ability to assess the costs and benefits of potential treatments based on the broader context of the patient’s lived environment [49].

There needs to be good role modelling in doctor-patient interaction in a variety of clinical settings. This entails establishing strong doctor-patient relations through communication with patients even though a preconceived idea about the latter’s conditions can be obtained from the EHRs. For instance, doctors can make use of EHRs to have a better understanding of patients’ conditions before their consultation sessions in order to have a more engaging session. Past studies have also shown that students and doctors can be trained to improve their communication skills and use EHRs in a patient-centred manner based on the content of the consultation, such as paying attention to the patient when he or she expresses a psychosocial issue [19, 50].

### Strengths and limitations

This study contributes to the growing body of international literature while specifically informing us about the concerns and challenges of integrating digital health into Singapore’s medical school curriculum. In view of the paucity of literature on the incorporation of digital competencies in Asian medical schools, it has evaluated the types of skills that would be relevant to the Singapore context and the ways in which a digital-oriented curriculum can be adapted to an Asian country that is experiencing a rapidly ageing population.

The sample achieved diversity in specialties, professional roles and types of healthcare institutions, which contributed to a rich data. By obtaining feedback from clinical educators and teachers, this study offers first-hand accounts of the current context of Singapore’s medical school curriculum before Singapore can embark on the integration of digital competencies into medical students’ education. Interviewing educators from all three local medical schools and doctors from different healthcare institutions ensures comprehensiveness of data and highlights the recurrent concerns among the respondents, suggesting that they are not merely unique to individual institutions but are commonly shared by many across the country.

A potential line of future research would be to explore the views of healthcare administrators and other stakeholders on other concerns and challenges that may have been missed by the respondents. The qualitative feedback obtained from this study could also be used to develop a checklist or questionnaire that identifies gaps for specific digital competencies.

## Conclusions

In conclusion, our study has shown that while Singapore is on par with many other developed countries such as the US in terms of digital competitiveness, its medical school curricula have to be suited to the socio-cultural needs of the population. Furthermore, while it is pertinent to keep up with technological advances, preserving basic clinical skills, the human touch and a broad-based curriculum are equally if not more important for medical students in Singapore. At the same time, this study bears important implications for other developed countries that are also evaluating the type of skills in digital technologies that should be imparted to medical students to better prepare them for clinical practice. It has demonstrated this by exploring the concerns of some senior clinical educators and teachers in Singapore. Specifically, it has shown that the main concerns behind the implementation of a digital-centric curriculum lie in a multitude of factors that stem from both new and existing concerns that are widely applicable. The findings have also called for a shift in how we view technology and the role of doctors. In essence, technology should complement instead of replace core clinical skills.

## Data Availability

The datasets generated and analysed during the current study are not publicly available to protect the confidentiality and anonymity of participants but are available from the corresponding author on reasonable request.

## Abbreviations

AI: Artificial Intelligence
ML: Machine learning
VR: Virtual reality
POCUS: Point-of-care ultrasound
EHRs: Electronic health record systems

## References

[1] IMD World Competitiveness Ranking 2020: showing strength of small economies | IMD News [Internet]. [Cited 2022 Mar 14]. Available from: https://www.imd.org/news/updates/IMD-2020-World-Competitiveness-Ranking-revealed/

[2] Embracing innovative tech and robots in healthcare - SingHealth [Internet]. [Cited 2022 Mar 14]. Available from: https://www.singhealth.com.sg/news/singapore-health/embracing-innovative-tech-and-robots-in-healthcare

[3] Yeoh K-G (2019). The future of medical education. Singapore Med J.

[4] The age of opportunity – Future Health Index report 2020 | Philips [Internet]. [Cited 2022 Mar 14]. Available from: https://www.philips.com.sg/a-w/about/news/future-health-index/reports/2020/the-age-of-opportunity.html

[5] Machleid F, Kaczmarczyk R, Johann D, Balčiūnas J, Atienza-Carbonell B, von Maltzahn F (2020). Perceptions of Digital Health Education Among European Medical Students: Mixed Methods Survey. J Med Internet Res.

[6] Stanford Medicine’s 2020 Health Trends Report spotlights the rise of the data-driven physician | News Center | Stanford Medicine [Internet]. [Cited 2022 Mar 14]. Available from: https://med.stanford.edu/news/all-news/2020/01/health-trends-report-spotlights-rise-of-data-driven-physician.html

[7] Wood EA, Ange BL, Miller DD (2021). Are We Ready to Integrate Artificial Intelligence Literacy into Medical School Curriculum: Students and Faculty Survey. J Med Educ Curric Dev.

[8] The Five Biggest Healthcare Tech Trends In 2022 [Internet]. [Cited 2022 Mar 14]. Available from: https://www.forbes.com/sites/bernardmarr/2022/01/10/the-five-biggest-healthcare-tech-trends-in-2022/?sh=750ab0fa54d0

[9] Cutrer WB, Spickard WA, Triola MM, Allen BL, Spell N, Herrine SK (2021). Exploiting the power of information in medical education. Med Teach.

[10] Paranjape K, Schinkel M, Nannan Panday R, Car J, Nanayakkara P (2019). Introducing Artificial Intelligence Training in Medical Education. JMIR medical education.

[11] Foadi N, Koop C, Mikuteit M, Paulmann V, Steffens S, Behrends M (2021). Defining Learning Outcomes as a Prerequisite of Implementing a Longitudinal and Transdisciplinary Curriculum with Regard to Digital Competencies at Hannover Medical School. J Med Educ Curric Dev.

[12] Casà C, Marotta C, di Pumpo M, Cozzolino A, D’Aviero A, Frisicale EM (2021). COVID-19 and digital competencies among young physicians: are we (really) ready for the new era? A national survey of the Italian Young Medical Doctors Association. Annali dell’Istituto superiore di sanita.

[13] Hautz SC, Hoffmann M, Exadaktylos AK, Hautz WE, Sauter TC (2020). Digital competencies in medical education in Switzerland: an overview of the current situation. GMS J Med Educ..

[14] Rienits H, Teuss G, Bonney A (2016). Teaching telehealth consultation skills. Clin Teach.

[15] Taylor B, Postuma P, Paterson G, Hurley KF. What are Canadian Medical Students Learning about Health Informatics. Journal of Health Informatics 6 np electronic Journal of Health Informatics www.eJHI.net [Internet]. 2011 [Cited 2022 Mar 14];6. Available from: www.eJHI.net

[16] Valikodath NG, Cole E, Ting DSW, Campbell JP, Pasquale LR, Chiang MF (2021). Impact of Artificial Intelligence on Medical Education in Ophthalmology. Transl Vis Sci Technol.

[17] Wartman SA (2019). The Empirical Challenge of 21st-Century Medical Education. Academic Med.

[18] Brockes C, Grischott T, Dutkiewicz M, Schmidt-Weitmann S (2017). Evaluation of the Education “Clinical Telemedicine/e-Health” in the Curriculum of Medical Students at the University of Zurich. Telemed J E health.

[19] Lee WW, Alkureishi ML, Wroblewski KE, Farnan JM, Arora VM (2017). Incorporating the human touch: piloting a curriculum for patient-centered electronic health record use. Med Educ Online.

[20] Tong A, Sainsbury P, Craig J (2007). Consolidated criteria for reporting qualitative research (COREQ): a 32-item checklist for interviews and focus groups. Int J Qual Health Care.

[21] O’Brien BC, Harris IB, Beckman TJ, Reed DA, Cook DA (2014). Standards for Reporting Qualitative Research. Acad Med.

[22] Kiger ME, Varpio L (2020). Thematic analysis of qualitative data: AMEE Guide No. 131. Med Teach..

[23] Braun V, Clarke V (2006). Using thematic analysis in psychology. Qual Res Psychol.

[24] Aungst TD, Patel R (2020). Integrating Digital Health into the Curriculum-Considerations on the Current Landscape and Future Developments. J Med Educ Curric Dev.

[25] Preparing the doctor of the future | Deloitte Insights [Internet]. [Cited 2022 Mar 14]. Available from: https://www2.deloitte.com/us/en/insights/industry/health-care/doctor-of-the-future-medical-school-residency-programs.html

[26] Han E-R, Yeo S, Kim M-J, Lee Y-H, Park K-H, Roh H (2019). Medical education trends for future physicians in the era of advanced technology and artificial intelligence: an integrative review. BMC Med Educ.

[27] Medical Grand Challenge [Internet]. [Cited 2022 Mar 14]. Available from: https://medicine.nus.edu.sg/cenmed/mgc/

[28] Zainal H, Smith HE (2020). Medical students’ attitudes towards careers in primary care in Singapore. BMC Med Educ.

[29] Outcomes and Standards for Undergraduate Medical Education in Singapore. 2014;

[30] Gonzalo JD, Dekhtyar M, Starr SR, Borkan J, Brunett P, Fancher T (2017). Health Systems Science Curricula in Undergraduate Medical Education: Identifying and Defining a Potential Curricular Framework. Acad Med.

[31] Lee-Barber J, Kulo V, Lehmann H, Hamosh A, Bodurtha J (2019). Bioinformatics for medical students: a 5-year experience using OMIM® in medical student education. Genet Med.

[32] Chong JA, Quah YL, Yang GM, Menon S, Radha Krishna LK (2015). Patient and family involvement in decision making for management of cancer patients at a centre in Singapore. BMJ Support Palliat Care.

[33] Garibaldi BT, Niessen T, Gelber AC, Clark B, Lee Y, Madrazo JA (2017). A novel bedside cardiopulmonary physical diagnosis curriculum for internal medicine postgraduate training. BMC Med Educ.

[34] Rousseau M, Könings KD, Touchie C (2018). Overcoming the barriers of teaching physical examination at the bedside: more than just curriculum design. BMC Med Educ.

[35] Paterick ZR, Patel NJ, Paterick TE (2018). Unintended consequences of the electronic medical record on physicians in training and their mentors. Postgrad Med J.

[36] Chiu H-Y, Kang Y-N, Wang W-L, Chen C-C, Hsu W, Tseng M-F (2019). The Role of Active Engagement of Peer Observation in the Acquisition of Surgical Skills in Virtual Reality Tasks for Novices. J Surg Educ.

[37] Ho AM-H, Critchley LAH, Leung JYC, Kan PKY, Au SS, Ng SK (2015). Introducing Final-Year Medical Students to Pocket-Sized Ultrasound Imaging: Teaching Transthoracic Echocardiography on a 2-Week Anesthesia Rotation. Teach Learning Med..

[38] Nomura T, Mamada Y, Nakamura Y, Matsutani T, Hagiwara N, Fujita I (2015). Laparoscopic skill improvement after virtual reality simulator training in medical students as assessed by augmented reality simulator. Asian J Endosc Surg.

[39] Aulenkamp J, Mikuteit M, Löffler T, Schmidt J (2021). Overview of digital health teaching courses in medical education in Germany in 2020. GMS J Med Educ..

[40] Kolachalama VB, Garg PS (2018). Machine learning and medical education. NPJ Digital Med.

[41] Ahn JS, French AJ, Thiessen MEW, Browne V, Deutchman M, Guiton G (2015). Using ultrasound to enhance medical students’ femoral vascular physical examination skills. J Ultrasound Med.

[42] Swamy M, Searle RF (2012). Anatomy teaching with portable ultrasound to medical students. BMC Med Educ.

[43] Benjamens S, Dhunnoo P, Meskó B (2020). The state of artificial intelligence-based FDA-approved medical devices and algorithms: an online database. NPJ digital medicine.

[44] Rijken M, Hujala A, van Ginneken E, Melchiorre MG, Groenewegen P, Schellevis F (2018). Managing multimorbidity: Profiles of integrated care approaches targeting people with multiple chronic conditions in Europe. Health Policy (Amsterdam, Netherlands).

[45] Søndergaard E, Willadsen TG, Guassora AD, Vestergaard M, Tomasdottir MO, Borgquist L (2015). Problems and challenges in relation to the treatment of patients with multimorbidity: General practitioners’ views and attitudes. Scand J Prim Health Care.

[46] Wartman SA, Combs CD (2018). Medical Education Must Move From the Information Age to the Age of Artificial Intelligence. Acad Med.

[47] Weiner M, Callahan CM, Tierney WM, Overhage JM, Mamlin B, Dexter PR (2003). Using information technology to improve the health care of older adults. Ann Intern Med.

[48] Haag M, Igel C, Fischer MR, German Medical Education Society (GMA) C “Digitization – T-AL and T, Joint working group “Technology-enhanced Teaching and Learning in Medicine (TeLL)” of the German Association for Medical Informatics B and E (gmds) and the GIS (GI) Digital Teaching and Digital Medicine: A national initiative is needed. GMS journal for medical education. 2018;35(3):Doc43. 10.3205/zma001189 PMC6120157 30186953

[49] Li D, Kulasegaram K, Hodges BD (2019). Why We Needn’t Fear the Machines: Opportunities for Medicine in a Machine Learning World. Academic Med.

[50] Lanier C, Dominicé Dao M, Hudelson P, Cerutti B, Junod PN (2017). Learning to use electronic health records: can we stay patient-centered? A pre-post intervention study with family medicine residents. BMC Fam Pract.

